# An improved solvent-free synthesis of flunixin and 2-(arylamino) nicotinic acid derivatives using boric acid as catalyst

**DOI:** 10.1186/s13065-017-0355-4

**Published:** 2017-12-01

**Authors:** Mahsa Yarhosseini, Shahrzad Javanshir, Zahra Dolatkhah, Mohammad G. Dekamin

**Affiliations:** Heterocyclic Chemistry Research Laboratory, Department of Chemistry, Iran University of Science and Technology, Tehran, 16846-13114 Iran

**Keywords:** Homogeneous catalysis, Solvent-free, Non-steroidal anti-inflammatory drugs (NSAID), *N*-Methyl-d-glucamine, Boric acid, Anilino-nicotinic acid

## Abstract

A simple solvent-free protocol for the preparation of flunixin, a potent non-narcotic, non-steroidal anti-inflammatory drugs is reported using boric acid as catalyst. Its salt, flunixin meglumine are then prepared under reflux in EtOH. This sustainable method are then extended for the synthesis of a series of 2-(arylamino) nicotinic acid derivatives. The present protocol combines non-hazardous neat conditions with associated benefits like excellent yield, straightforward workup, and use of readily available and safe catalyst in the absence of any solvent, which are important factors in the pharmaceutical industry. The pathway for catalytic activation of 2-chloronicotic acid with boric acid was also investigated using Gaussian 03 program package.

**Electronic supplementary material:**

The online version of this article (10.1186/s13065-017-0355-4) contains supplementary material, which is available to authorized users.

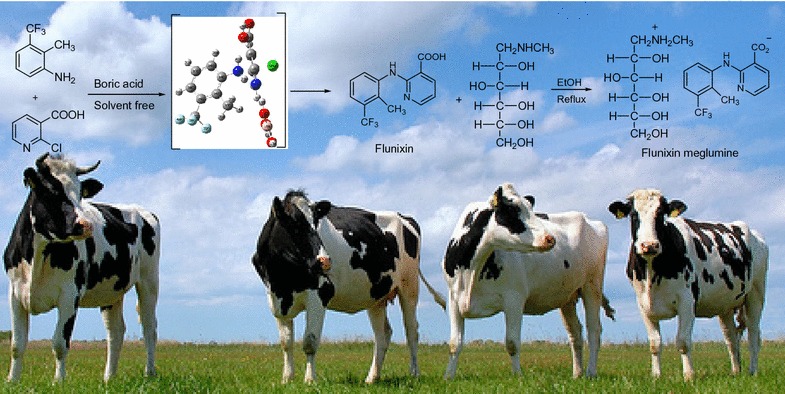

## Introduction

**Fig. 1:**
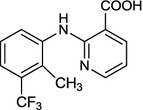
Chemical structure of flunixin

Flunixin, 2-(2-methyl-3-trifluoromethylanilino)nicotinic acid (Fig. 1) is a non-narcotic, non-steroidal anti-inflammatory drug (NSAID) and cyclooxygenase inhibitor with potent antipyretic and analgesic activity, parenterally administered in clinical and veterinary applications [1–3]. It is frequently administered in the form of the meglumine salt.

The analgesic activity of *N*-methyl-d-glucamine salt of flunixin (flunixin meglumine) is comparable to that observed by powerful narcotic analgesics such as morphine, meperidine hydrochloride, and pentazocine. However, the undesirable side effects are less compared to the above mentioned narcotics analgesics and drug dependence liability does not occur [4]. Apart from its parenteral analgesic activities, flunixin meglumine also possesses anti-inflammatory attributes [5–8]. Flunixin meglumine was first reported in 1975 [9] and is the active pharmaceutical ingredient in numerous drug products such as Resflor, Banamine Solution, Banamine Paste, Banamine Granules and Finadyne [10–19].

So far, several improved methods have been developed for the synthesis of flunixin including classical reflux in water [12], xylene [5] and ethylene glycol [20]. Some of these methods suffer from several drawbacks such as difficult workup, long reaction times, use of large quantities of non-green organic solvents such as xylene that are harmful for environment and harsh reaction conditions. In this regard, solvent-free approach which has become increasingly popular in recent years for reasons of economy and pollution prevention, as well as cleaner products, simple work-up, high-speed due to the high concentration of materials, and excellent yields would be the ideal approach, as it is often claimed that “the best solvent is no solvent” [21]. To the best of our knowledge, we herein report the first green synthesis of 2-(2-methyl-3-trifluoromethylanilino)nicotinic acid (**3a**) under solvent-free conditions in the presence of catalytic amount of Boric acid. In this contribution, studies to reach a broader scope and generality of this reaction is also presented using other anilines, as nucleophiles, some 2-arylaminonicotinic acids derivatives **3** were also synthesized by this method.

## Results and discussion

**Scheme 1:**
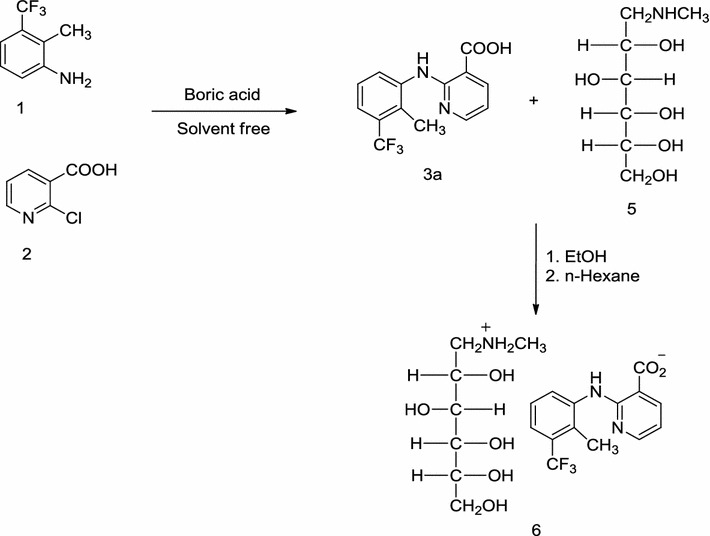
Synthesis of flunixin meglumine

Boric acid [H_3_BO_3_ or B(OH)_3_] has attracted particular attention in recent years as catalyst in organic synthesis [22–25], because of many advantages such as uncomplicated handling, inexpensiveness, eco-friendly nature and commercially available [26]. Consequently, we have considered the synthesis of 2-(2-methyl-3-trifluoromethylanilino)nicotinic acid (**3a**), in the presence of boric acid under solvent-free conditions, and its salt, flunixin meglumine (**6**) under reflux in EtOH (Scheme 1).

In a first attempt, the reaction of 2-methyl-3-trifluoromethylanilin (**1**) with 2-chloronicotinic acid (**2**) was examined under various reaction conditions, the results and the optimization of reaction conditions are summarized in Table 1. Initially, the effect of acid and basic catalysts was tested. In the absence of any catalyst no reaction took place under reflux in water (Table 1, entry 1), and also under solvent-free condition the desired product was obtained in low yield in the absence of any catalyst (entry 2). When this reaction is carried out in the presence of a catalyst such as K_2_CO_3_, NEt_3_, Fe_3_O_4_, and DABCO in the presence and absence of solvent only a trace amount of product was detected even after 24 h (entries 5–8), while the desired product was obtained in moderate yield in the presence of PTSA and boric acid (entries 9 and 10). Considering the cost and toxicity of these two catalysts, and also due to a slight difference in efficiency between them (entries 21 and 23), we choose boric acid as optimal catalyst.

**Table 1:** Optimization of the reaction conditions for the synthesis of (**3a**)

Entry	Ratio of 1/2	Catalyst	Catalyst loading (mole %)	Solvent	Temp (°C)	Time (h)	Yield^c^ (%)
1	2:1	–	–	H_2_O	Reflux	24	–
2	2:1	–	–	–	100	10	35
3	2:1	K_2_CO_3_	15	–	100	24	Trace
4	2:1	NEt_3_	15	–	100	24	–
5	2:1	K_2_CO_3_	15	H_2_O	Reflux	24	Trace
6	2:1	NEt_3_	15	H_2_O	Reflux	24	–
7	2:1	Fe_3_O_4_	15	H_2_O	Reflux	24	–
8	2:1	DABCO^a^	15	H_2_O	Reflux	24	Trace
9	2:1	PTSA	15	H_2_O	Reflux	24	65
10	2:1	H_3_BO_3_	15	H_2_O	Reflux	24	60
11	2:1	H_3_BO_3_	15	EtOH	Reflux	24	40
12	2:1	H_3_BO_3_	15	*n*-Hexanol	Reflux	24	62
13	2:1	H_3_BO_3_	15	PEG-400^b^	Reflux	24	30
14	2:1	H_3_BO_3_	15	DMF	Reflux	24	45
15	2:1	H_3_BO_3_	15	Xylene	Reflux	24	Trace
14	2:1	H_3_BO_3_	15	Toluene	Reflux	24	50
17	2:1	H_3_BO_3_	15	–	100	10	68
18	2:1	H_3_BO_3_	30	–	100	10	82
19	2:1	H_3_BO_3_	48	–	100	10	84
20	2:1	H_3_BO_3_	30	–	80	12	45
*21*	2:1	*H*_*3*_ *BO*_*3*_	*30*	–	*120*	*10*	*90*
22	2:1	H_3_BO_3_	30	–	150	10	90
23	2:1	PTSA	30	–	120	9	90
24	1:1	H_3_BO_3_	30	–	120	20	60

Reaction conditions: 2-methyl-3-trifluoromethylanilin (2 mmol), 2-chloronicotinic acid (1 mmol)

The optimum reaction conditions are in italics

^a^1,4-diazabicyclo[2.2.2]octane

^b^Polyethylene glycol

^c^The yields refer to the isolated product

In the next step the effect of various solvents and temperature (80–150 °C) was examined (Table 1, entries 8–22). The best yield was obtained at 120 °C under solvent-free condition (entry 17). Therefore, considering the viewpoints of green chemistry, the synthesis of flunixin was carried out under solvent-free condition. And finally, we studied the effect of the ratio of reactants on the yield of the reaction. When the reagents were used in an equimolar ratios, a reduction in yield was observed (entry 24) and the optimum ratio of 2-methyl-3-trifluoromethylanilin (**1**) to 2-chloronicotinic acid (**2**) was 2:1 (entry 21).

**Scheme 2:**
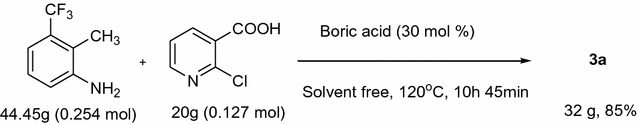
Scalability of the reaction to the multi-gram scale

Therefore, the optimum reaction conditions for the synthesis of flunixin were as follows: molar ratio of 2-methyl-3-trifluoromethylanilin (**1**) to 2-chloronicotinic acid (**2**) equal to 2:1 at 120 °C under solvent-free condition in the presence of H_3_BO_3_ (20 mg, 30 mol%) as catalyst (Table 1, entry 21). This procedure was also scaled up to 30 g of product and the yield of flunixin was maintained excellent however the reaction time was augmented about 45 min (Scheme 2).

The synthesized flunixin was characterized by FT-IR, ^1^HNMR, ^13^CNMR and GC/MS (see Additional file 1). A quantitative analysis was performed to determine the amount of residual boron in the synthesized flunixin. The result of ICP analysis shows a level of 2.23 ppm (2.23 mg/L) of boron in the product. According to the World Health Organization, the health-based guideline for boron level in drinking water is 2.4 mg/L.

Since we have been very successful in this solvent free approach with a very specific aromatic amine such as 2-methyl-3-trifluoromethylanilin, we decided to study the scope and limitation of the reaction using other aniline derivatives as nucleophiles. Reactions of aniline derivatives with 2-chloronicotinic acid gave good-to-excellent yields. This result was expected because the pyridine ring is activated toward nucleophilic attack by the formation of a pyridinium salt with the acid catalyst. Table 2 shows several 2-arylaminonicotinic acids derivatives synthesized under solvent-free condition in the presence of 20 mg H_3_BO_3_ at 120 °C. As it can be seen, nucleophilic substitution takes place readily at the 2- and 4-positions of a ring particularly when substituted with an effective leaving group such as a halogen atom. In nucleophilic substitution, the intermediate is negatively charged.

**Table 2:** Results of the synthesis of 2-arylaminonicotinic acids derivatives **3**(**b**–**l**) under solvent-free conditions

					
Entry	Anilines	Products	Time (min)	Yield^a^ (%)	Mp (°C)
1	2-Chloroaniline	**3b**	20	62	213–215 [212–214] [28]
2	2,6-Dichloroaniline	**3c**	60	25	264–267 [266–267] [29]
3	3,4-Dichloroaniline	**3d**	25	72	258–260 [260] [30]
4	2,3-Dichloroaniline	**3e**	25	58	256 [256–258] [5]
5	2,4-Dimethylaniline	**3f**	10	92	225–227 [225] [31]
6	3-Nitroaniline	**3g**	30	90	218–219 [216–218] [32]
7	4-Nitroaniline	**3h**	60	28	274–275 [272–274] [29]
8	1-Naphthylamine	**3i**	30	85	191–194 [193–194] [33]
9	2-Aminophenol	**3j**	25	88	230–232 [230–232] [34]
10	Methylamine	**3k**	–	–	–
11	Buthylamine	**3l**	–	–	–
12	Phenylethylamine	**3m**	–	–	[228–230] [35]

Reaction conditions: amines (2 mmol), nicotinic acid (1 mmol), and 20 mg H_3_BO_3_ at 120 °C under solvent-free condition

^a^The yields refer to the isolated product

**Scheme 3:**
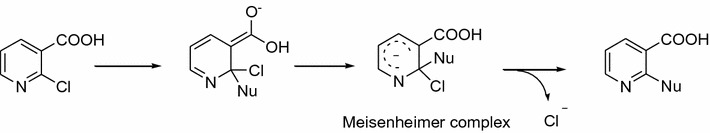
Nucleophilic substitution of a pyridine ring

The capacity of the ring to withstand the negative charge determines the stability of the intermediate and transition state which drives it and consequently determines the reaction rate. Nucleophilic attack at the 2-position yields a carbanion that is hybrid of structures. The hybrid structures of the anionic intermediate formed during the nucleophilic attack are especially stable, since the negative charge is located on the electronegative nitrogen atom which can better accommodate it (Scheme 3). For this reason the nucleophilic substitution occurs specially on the pyridine ring instead of the benzene ring and preferably at the 2 and 4 positions.

Various anilines were tolerated in this methodology and gave the product in good yield (Table 2, entries 1, 3, 4, 5, 6, 8, 9), but some anilines (entry 2 and 7) evince the influence of electron withdrawing group and steric hindrance on their reactivity by giving a low product yield. Remarkably, when primary amines other than anilines were selected for this transformation, no reaction was observed and they are not potent nucleophiles in this methodology; this could be attribute to the higher nucleophilicity of the acyclic primary amines poisoning the catalyst as a result of competitive binding with the primary amine to the boric acid. The scope in substrates for nucleophilic substitution was also investigated using some pyridines derivatives such as 4-chloro pyridine, 3-chloro pyridine and 2-chloro-5-nitro pyridine. However, when the reaction was run using these substrates, no product was observed even after 12 h (Table 3). The effect of other substituents on the pyridine ring show that the substitution reactions are more facile if an activating group is present on the ring. 2-chloronicotinic acid contains a strongly electron-withdrawing group –COOH. This would be expected to better activate the halogen located *ortho* to it. This result clearly show the activating effect of the carboxylic acid group in S_N_Ar of aromatic ring by forming resonance-stabilized intermediate known as Meisenheimer complex [27].

**Table 3:** Scope in pyridine analogues for nucleophilic aromatic substitution

Entry	Substrate	Nucleophile	Time (h)	Yield (%)
1			12	–
2			12	–
3			12	–

Reaction conditions: aniline (2 mmol), pyridine analogues (1 mmol), and 20 mg (0.3 mmol) H_3_BO_3_ at 120 °C under solvent-free condition

The results for synthesis of 2-arylaminonicotinic acids derivatives **3** are very satisfactory, affording the respective products in good yield in short reaction times.

**Fig. 2:**
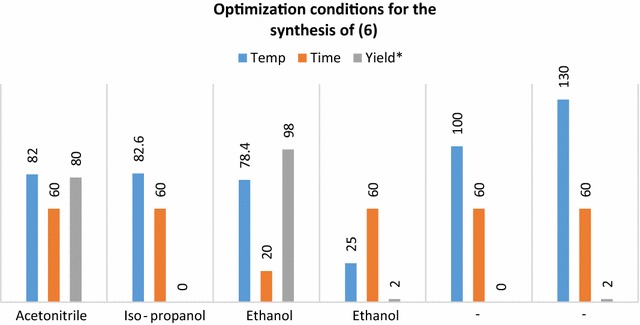
Optimization conditions for the synthesis of (**6**). Reaction conditions: flunixin (1 mmol), meglumine (1 mmol). *The yields refer to the isolated product

Flunixin is not soluble in water, and as it is administered by intravenous or intramuscular injection consequently to increase its solubility in water, it is often formulated as the meglumine salt. Therefore the reaction of flunixin (1 mmol) with meglumine (1 mmol) was also examined under various reaction conditions and the results and the optimization of reaction conditions are summarized in Fig. 2.

As it can be seen, refluxing in ethanol gave the best results among all examined conditions. The as produced flunixin meglumine was characterized by FTIR, ^1^HNMR, ^13^CNMR, GC/Mass (available in Additional file 1) and high-performance liquid chromatographic (HPLC) method was used to determine the purity to flunixin meglumine (see Additional file 1). As can be seen from the HPLC chromatogram, the purity of the synthesized flunixin meglumine is higher than the standard.

### Theoretical study of mechanism

The pathway for catalytic activation of 2-chloronicotic acid with Boric acid was investigated using theoretical gas-phase calculations [36]. The full geometry optimizations and property calculations were performed within the density functional theory (DFT) approach using the Becke’s three-parameter B3LYP exchange–correlation functional [37] and the 6-311G** basis set [38, 39].

**Fig. 3:**
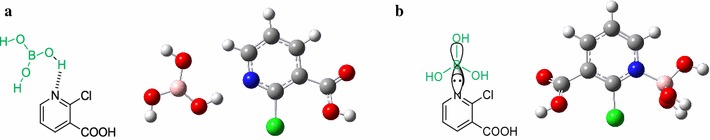
Two possible pathway for catalytic activation of 2-chloronicotic acid with Boric acid. **a** HBD, **b** Boron-Nitrogen Lewis acid–base interaction

**Fig. 4:**
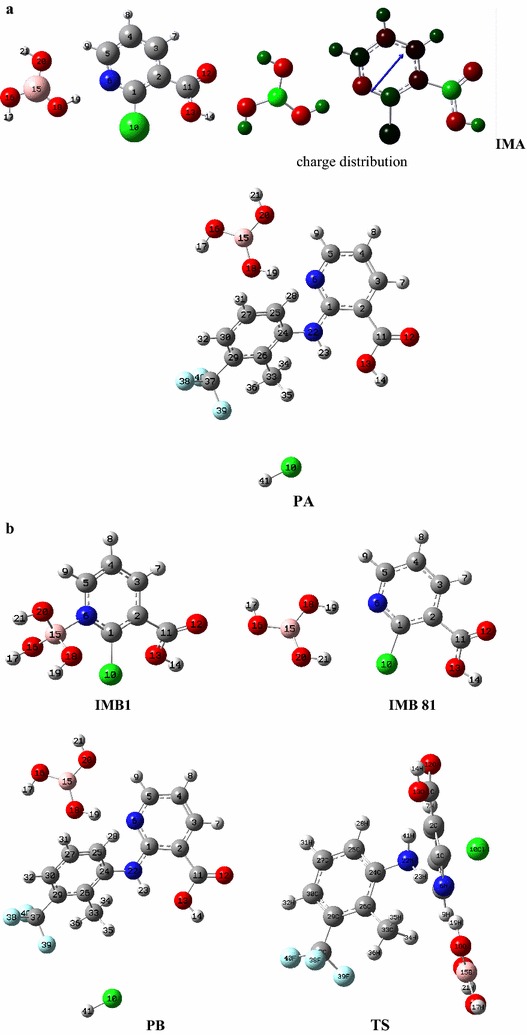
The optimized structures of nicotinic acid-boric acid, IMs, and product for pathway (**a**, **b**) by B3lyp/6-311G (d, p) singlet in the gas phase

The two possible pathway for catalytic activation of 2-chloronicotic acid with Boric acid, i.e. hydrogen bonding and lone pair co-ordinate bond with the empty orbital on the boron are presented in Fig. 3. The optimized geometries of IMs, products (P) and transition state (TS) and the numbering used in the analysis of the results are displayed in Fig. 4. The optimized bond lengths (in nm) and stability energies E (a.u.) for IMs, TS and products are tabulated in Table 4.

**Table 4:** The optimized bond lengths (in nm) and stability energies E (a.u.) computed at B3LYP/6-311G (d, p) level of theory for IMA, IMB, TS, PA, and PB (see Fig. 4 for atoms numbering)

Bond length (nm)	IMA	IMB	TS	PA	PB
(N6–H19)	1.92	1.95		1.92	1.92
(C1–Cl10)	1.75	1.75	2.04		
(C1–N22)			1.91	1.37	1.37
E (stability energies in a.u.)	− 1149.199 a.u.	− 1149.193 a.u.		− 1352.544 a.u.	− 1352.544 a.u.

The calculations overall indicate small affinity of boron for the nitrogen donor atom (IMB1) so that the optimized structure tend to form hydrogen bonds (HBDs) in (IMB 81) and give strong support to the suggested mechanism in which HBD formation between nitrogen in pyridine and OH of Boric acid is the preferred mode of activation. These results also showed that the inter-nuclear distance C1–Cl10 increased from 1.75 in IMA to 2.04 in TS indicating the cleavage of this bonds while the inter-nuclear distance N22–C1 decreased from 1.91 in TS to 1.37 (N21–C1) in PA. These observations provide evidence that the reaction proceeds through an addition elimination mechanism.

**Scheme 4:**
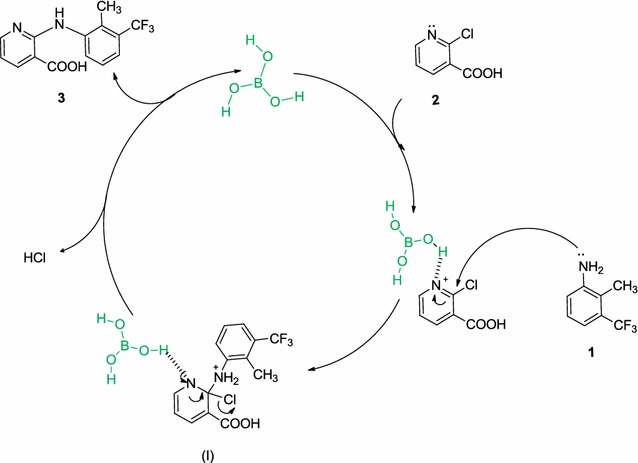
Plausible mechanisms for the synthesis of flunixin

We also used Gaussian 03 [36] to observe the vibrations in the TS and saw that the negative frequency corresponded to the motion of N22 and Cl10. According to obtained results, the following proposed mechanism seems reasonable for the synthesis of flunixin catalyzed by boric acid (Scheme 4). The substitution reactions proceed via an addition–elimination mechanism. In this proposed mechanism the nucleophilic aromatic substitution reaction occurs via a two-step mechanism in which the first step is the addition

2-methyl-3-trifluoromethylanilin (**1**) to the pyridine ring of 2-chloronicotinic acid (**2**) which was activated through HBD between pyridine and Boric acid and so leading to the intermediate (**I**). In the second step elimination of the halide ion restores the aromaticity leading to the product (**3a**).

## Conclusion

In summary, we have developed a simple, convenient and efficient method for the synthesis of flunixin using H_3_BO_3_ as catalyst under solvent-free conditions. This method are then extended for the synthesis of a series of 2-(arylamino)nicotinic acid derivatives. The present protocol has several advantages, particularly solvent- free conditions, high yields, ecofriendly operational, experimental simplicity along with mild conditions and the most important of them is development and optimization of flunixin meglumine synthesis in order to transfer to a larger scale for manufacture. Density function UB3LYP/6-311++g(d,p) calculations give strong support to the suggested mechanism in which HBD formation between nitrogen in pyridine and OH of Boric acid is the preferred mode of activation.

## Additional file

**Additional file 1.** Supporting Information.
