# Supplementary material for: An improved solvent-free synthesis of flunixin and 2-(arylamino) nicotinic acid derivatives using boric acid as catalyst
Source: Chem Cent J. 2017 Dec 1;11:124. doi: 10.1186/s13065-017-0355-4 (PMC5711764; doi:10.1186/s13065-017-0355-4)
Supplement: Supplementary file 1 — Additional file 1. Supporting Information. [file 13065_2017_355_MOESM1_ESM.docx]

**Supporting Information**

**An Improved Solvent-free Synthesis of Flunixin, 2-(arylamino) Nicotinic Acid Derivatives Using Boric Acid as Catalyst**

*Mahsa Yarhosseini^a^, Shahrzad Javanshir*^a^, Zahra Dolatkhah^a^, Mohammad G. Dekamin^a^*

^a^ Heterocyclic chemistry Research Laboratory, Department of Chemistry, Iran University of Science and Technology,Tehran 16846-13114, Iran. E-mail: [shjavan@iust.ac.ir](mailto:shjavan@iust.ac.ir)

| Contents | Page |
| --- | --- |
| Experimental section | 2 |
| 1. Instruments and characterization | 2 |
| 1. General Procedures for the synthesis of flunixin and flunixin meglumine | 2-3 |
| 1. Chemical characterization of flunixin **(3a)** | 3-7 |
| 1. Chemical characterization of flunixin meglumine **(6)** | 7-12 |

**Experimental section**

1. **Instruments and characterization**

All chemicals were purchased from Merck, Fluka and Sigma-Aldrich companies and were used without further purification. Analytical thin layer chromatography (TLC) for monitoring reactions was performed using Merck 0.2 mm silica gel 60 F-254 Al-plates using ethyl acetate and n-hexane as eluents. Melting points were determined in open capillaries using an Electrothermal 9100 instrument. Infrared (IR) spectra were acquired on a Shimadzu FT-IR-8400S spectrometer. ^1^H NMR (300 MHz) and ^13^C NMR (125 MHz) spectra were recorded on a Bruker DRX-500 Avance spectrometers with CDCl_3_ & DMSO-*d_6_* as solvents and tetramethylsilane (TMS) as the internal standard. All chemical shifts are given relative to TMS. All yields refer to the isolated products. HPLC analysis was performed using a Kenower model; column size: l = 0.125 m, Ø = 4.0 mm; stationary phase: octadecylsilyl silica gel for chromatography R (5µm); the mobile phase consisted of a mixture of 300 volumes water R and 700 volumes acetonitrile R, and 0.25 volumes of phosphoric acid R; the flow rate was 1.0 mL/min and UV detector spectrophotometer at 254 nm. GC/Mass analysis was performed using a Perkin-Elmer Clarus SQ 8S. Quantitative boron analysis were performed with a Shimadzu ICPS- 7000 ver.2.

1. **General Procedures**

*2.1. General procedure for the synthesis of flunixin* ***(3a)***

A mixture of 2-methyl-3-trifluoromethylanilin **(1)** (0.350 g, 2 mmol) and
2-chloronicotinic acid **(2)** (0.157 g, 1 mmol) was heated and stirred at 120°C for 10 hours in the presence of 20 mg boric acid (30 mol %) as catalyst. After the completion of the reaction monitored by TLC (ethylacetate:n-hexane, 2:1), the pH of reaction mixture was adjusted to 11 with NaOH 5% (W/V), then treated with activated charcoal (7 mg) and clarified after filtration. The filtrate was adjusted to pH=5 with concentrated sulfuric acid. The filtration of the precipitate gives 2-(2-methyl-3-trifluoro methylanilino) nicotinic acid (flunixin) as a white solid (0.24 g, 90% yield).

- 1. *General procedure for the synthesis flunixin meglumine* ***(6)***

A mixture of 2-(2-methyl-3-trifluoromethylanilino) nicotinic acid **(3a)** (0.296 g, 1mmol) and *N*-methyl-*D*-glucamine (Meglumine) (**5**) (0.195 g, 1mmol) were refluxed in 4 ml ethanol. After the completion of reaction as monitored by TLC (ethylacetate:n-hexane, 2:1), the reaction mixture was cooled to room temperature and n-hexane (4-5 ml) was subsequently added and the precipitated crystalline product was collected by filtration and dried to obtain 0.49 g of Flunixin meglumine as a white crystalline product (isolated yield 98%).

1. **Chemical characterization of flunixin (3a)**

White crystals, mp 223-225°C, isolated yield: %92.

IR (KBr) cm^-1^: 3238, 2300-2775, 2453, 1677, 1579, 1508, 1454, 1319, 1240, 1164, 1122, 1020, and 792. ^1^H NMR (300 MHz, DMSO-*d_6_*) δ (ppm): 2.34 (s, 3H, Me), 6.87 (dd, 1H, *J*= 7.5 and 4.8 Hz, Pyrid-H), 7.34-7.41 (m, 2H, Arom-H), 8.25 (d, 1H, *J*= 7.8 Hz, Pyrid-H), 8.32 (d, 1H, *J*=4.5, Arom-H), 8.37 (d, 1H, *J*= 7.5 Hz, Pyrid-H), 10.32 (s, 1H, -NH) and 13.51 (brs, 1H, -COOH). ^13^C-NMR (125 MHz CDCl_3_ & DMSO-*d_6_*) δ (ppm): 13.1, 107.4, 113.11, 119.7, 123.5, 125.00, 125.08, 125.7, 127.3 (m, CF_3_), 139.1, 140.0, 151.8, 155.6, 169.0.

C_14_H_11_F_3_N_2_O_2_, MW= 296/24 gr.mol^-1^. M/Z (%): 296(M+), 282, 139, 121, 91.


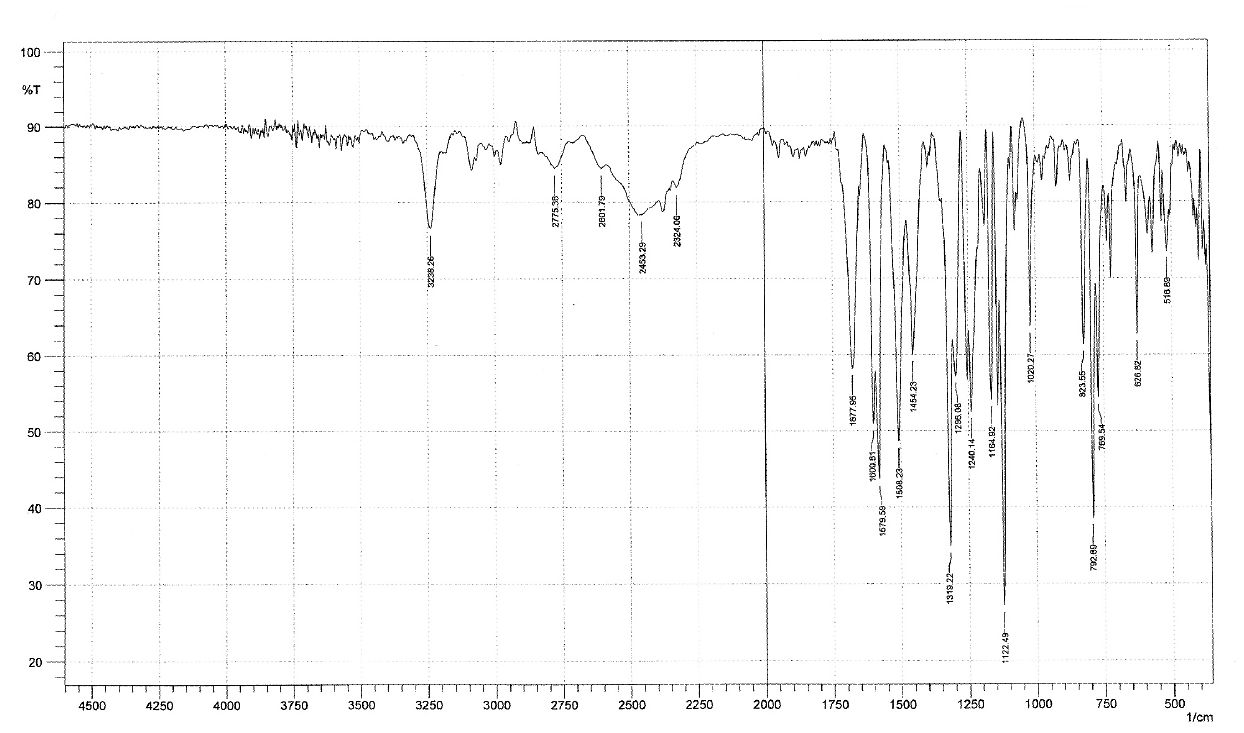

**Figure S-1.** FTIR spectrum of flunixin **(3a)**


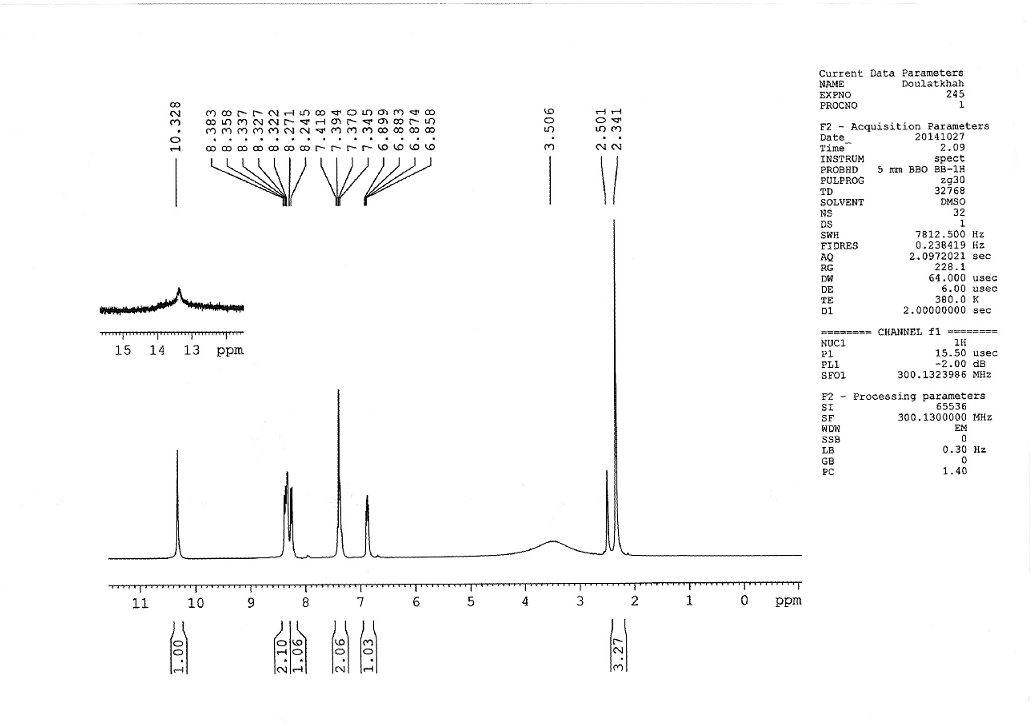

**Figure S-2.** ^1^HNMR spectrum of flunixin **(3a)** in DMSO-*d_6_*


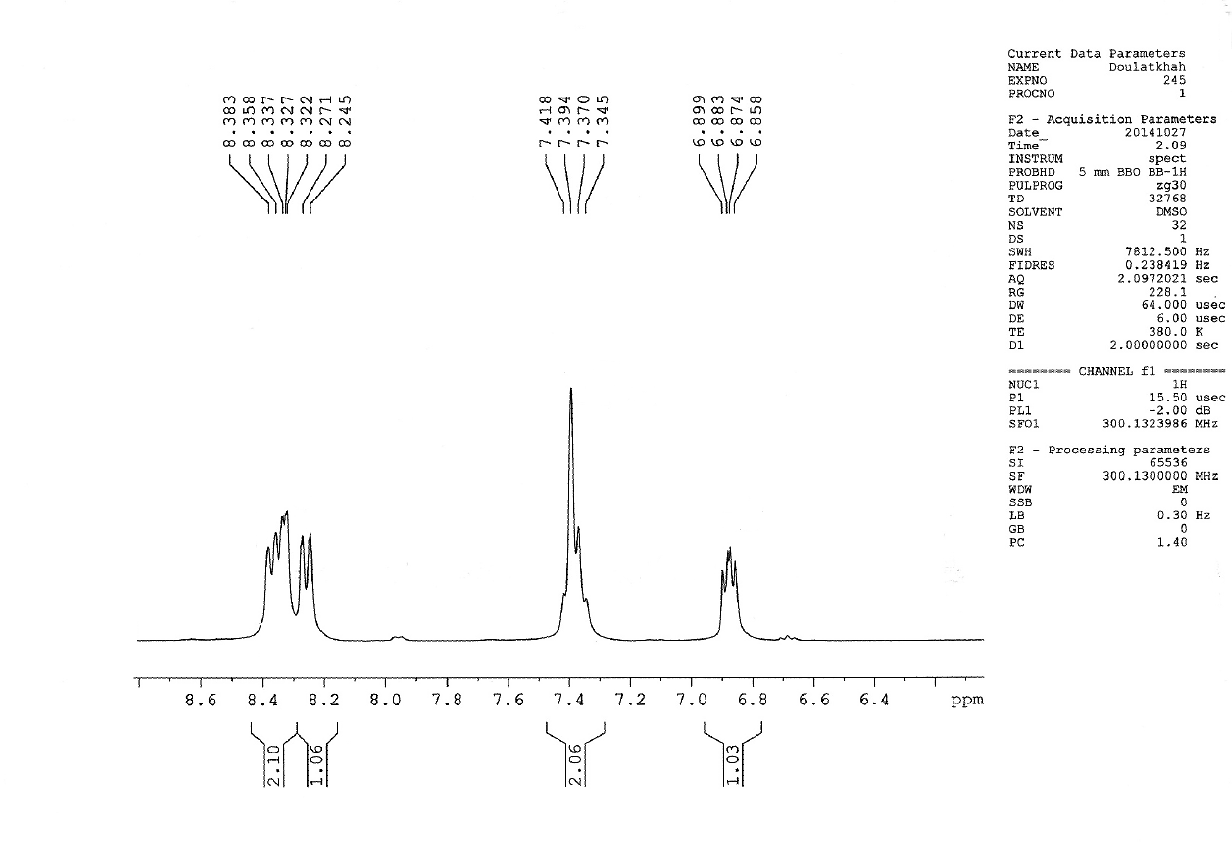

**Figure S-3.** ^1^HNMR spectrum of flunixin **(3a)** in DMSO-*d_6_* (expanded aromatic region)


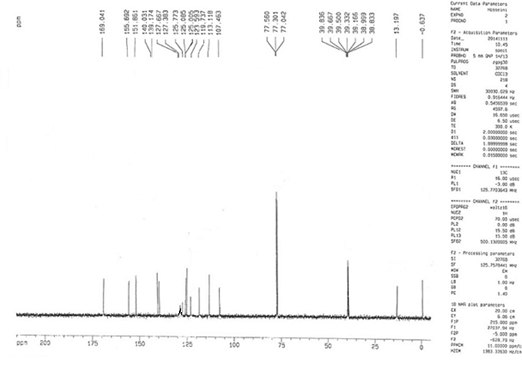

**Figure S-4.** ^13^C-NMR spectrum of flunixin **(3a)** in CDCl_3_ & DMSO-*d_6_*


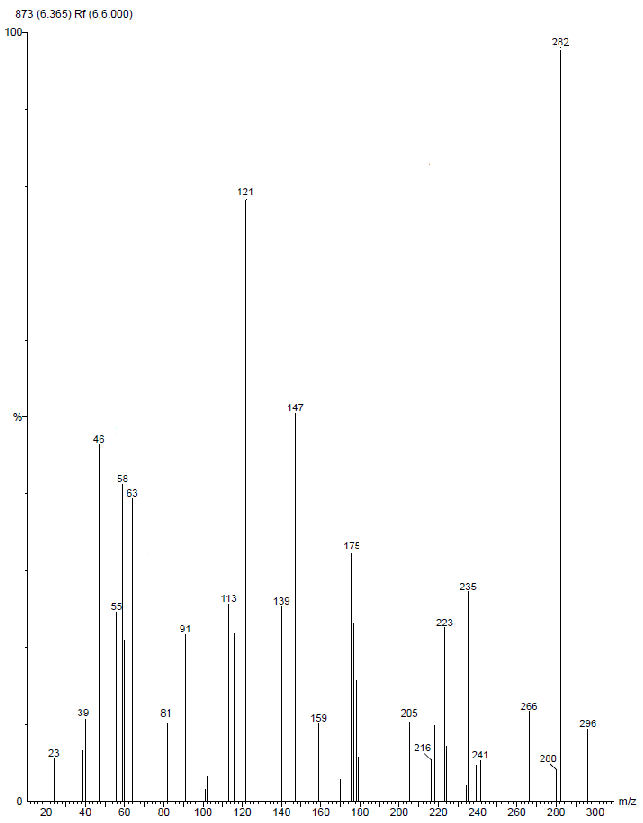

**Figure S-5.** GC/Ms spectrum of flunixin **(3a)** in 6.365 minutes


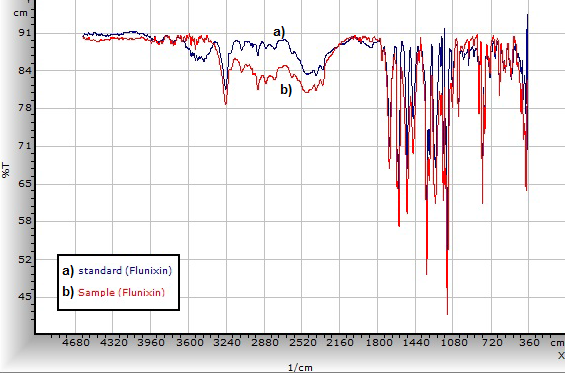


Synthesized flunexin

Standrad flunexin

**Figure S-6.** FT-IR spectrum of Standard and synthesized flunixin

The FT-IR of **3a** was compared with the standard sample (figure S-6) and shows that there is a perfect match between the standard and synthesized flunixin.

1. **Chemical characterization of flunixin meglumine (6)**

White crystals, mp 136-138 °C, yield: %98.

IR (KBr) cm^-1^: 3000-3400, 1587, 1384, 1120, and 1087. ^1^H NMR (300 MHz, DMSO-*d_6_*) δ (ppm): 2.39 (s, 3H, Arom-Me), 2.58 (s, 3H, -NCH_3_), 2.98 (m, 1H, Glucam-H), 3.10 (m, 1H, Glucam-H), 3.47 (m, 4H, Glucam-H), 3.61 (m, 1H, Glucam-H), 3.70 (m, 1H, Glucam-H), 3.95 (m, 2H, -NH_2_^+^), 4.76 (brs, 3H, -OH), 5.88 (brs, 2H, -OH), 6.78 (dd, 1H, J=7.5 and 4.8 Hz, Pyrid-H), 7.23 (d, 1H, J= 7.8 Hz, Arom-H), 7.32 (t, 1H, J= 7.8 Hz, Arom-H), 8.16 (dd, 1H, J= 8 Hz, Pyrid-H), 8.23 (dd, 1H, J=7.5 and 2.1 Hz, Pyrid-H), 8.80 (d, 1H, J=8.1, Arom-H), 12.56 (s, 1H, NH). ^13^C-NMR (125 MHz CDCl_3_ & DMSO-*d_6_*) δ (ppm): 13.7, 33.2, 51.7, 63.3, 68.6, 70.2, 71.2, 71.3, 106.4, 113.4, 115.1, 123.5, 125.3, 125.5, 126.3, 134.1 (m, CF_3_), 139.9, 140.9, 148.9, 155.7, 171.7.

C_21_H_28_F_3_N_3_O_7_, MW= 491/46 gr.mol^-1^, M/Z (%): 297, 251, 195.


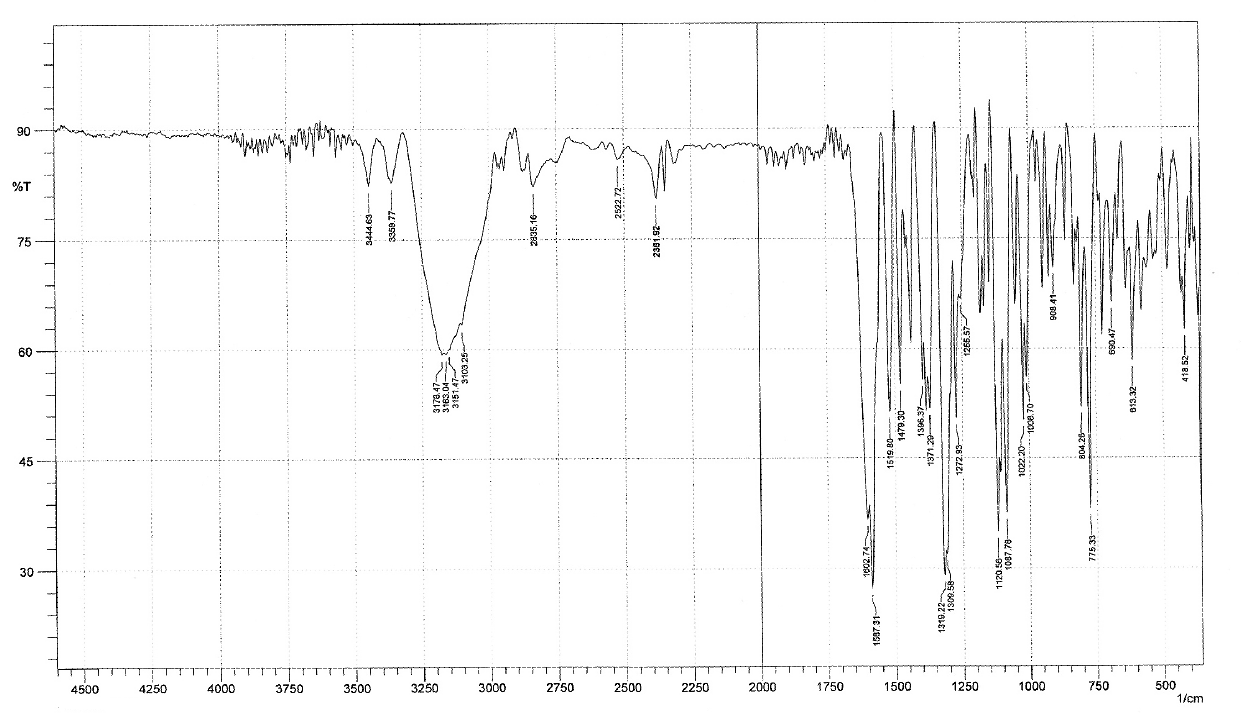

**Figure S-6.** FTIR spectrum of Flunixin meglumine **(6)**


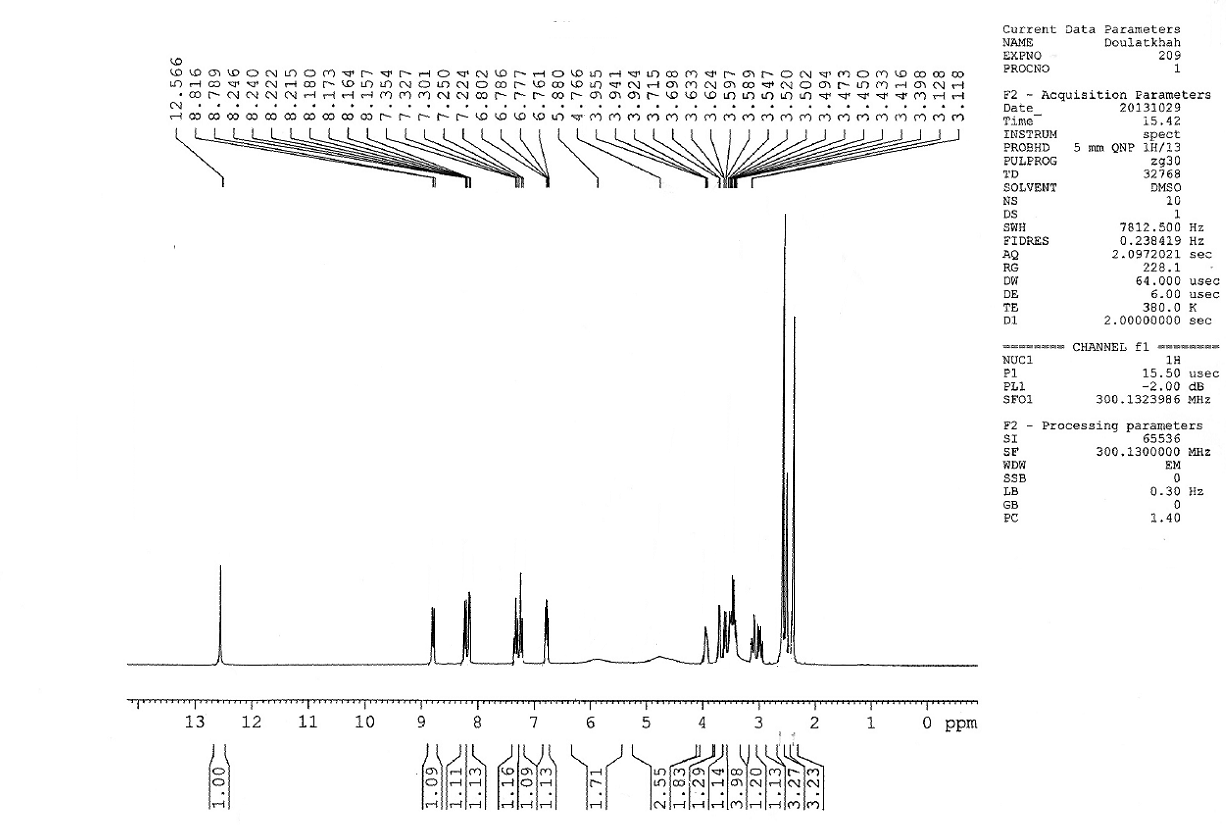

**Figure S-7.** ^1^HNMR spectrum of flunixin meglumine **(6)** in DMSO-*d_6_*


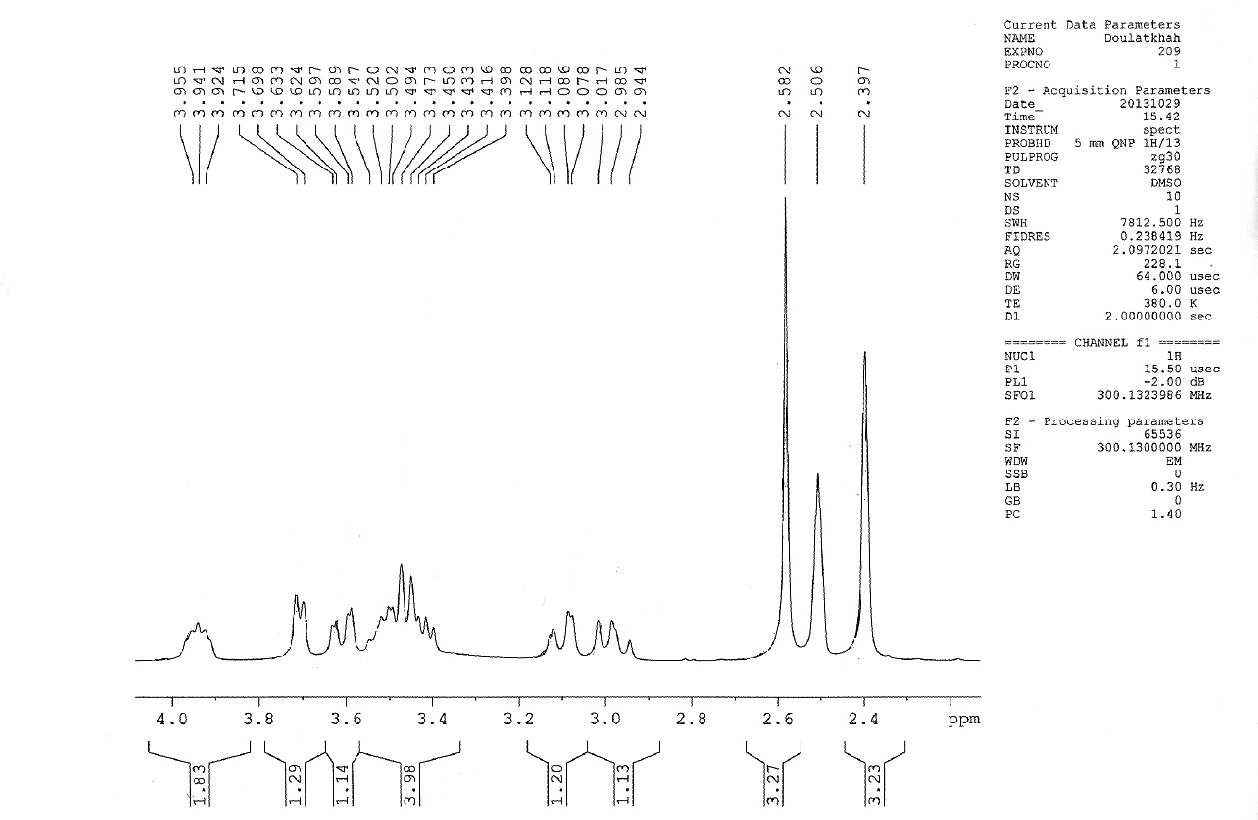

**Figure S-8.** ^1^HNMR spectrum of funixin meglumine **(6)** in DMSO-*d_6_* (Expanded aliphatic region)


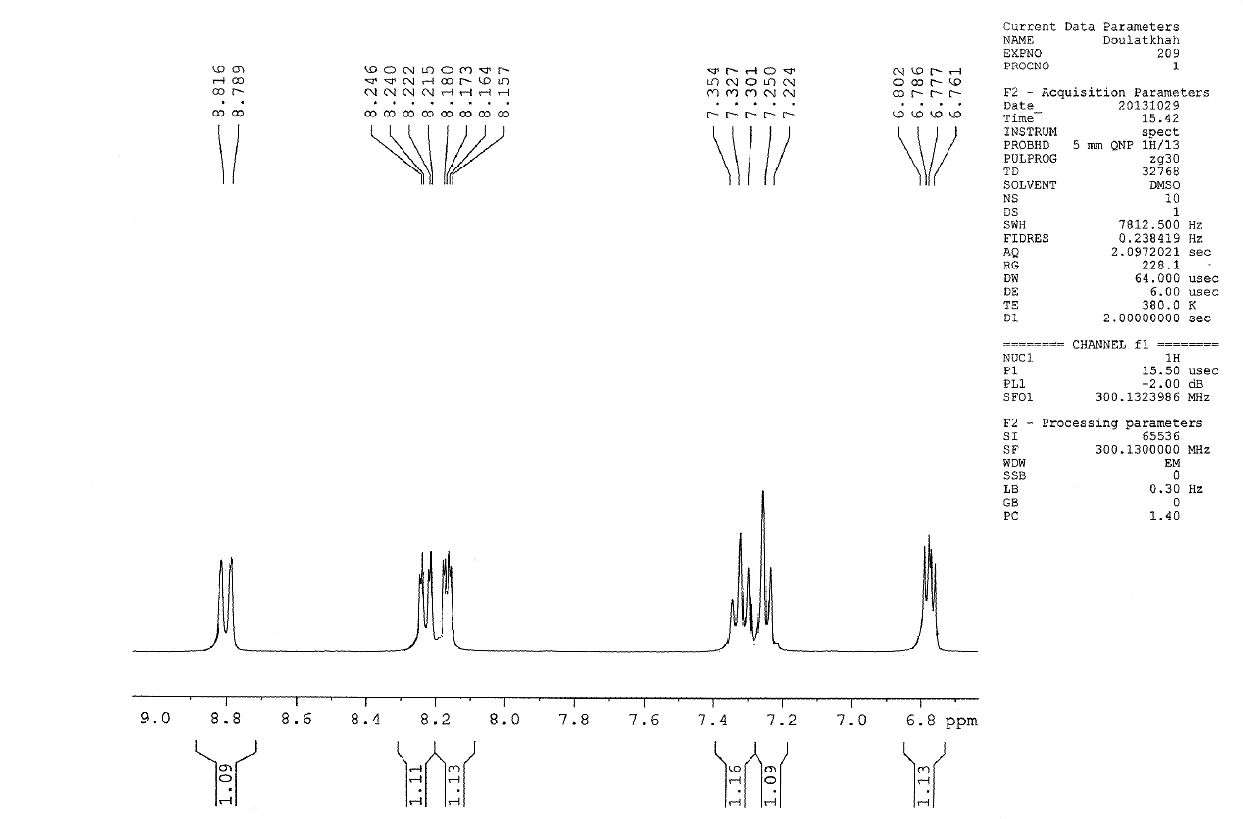

**Figure S-9.** ^1^HNMR spectrum of flunixin meglumine **(6)** in DMSO-*d_6_* (Expanded aromatic region)


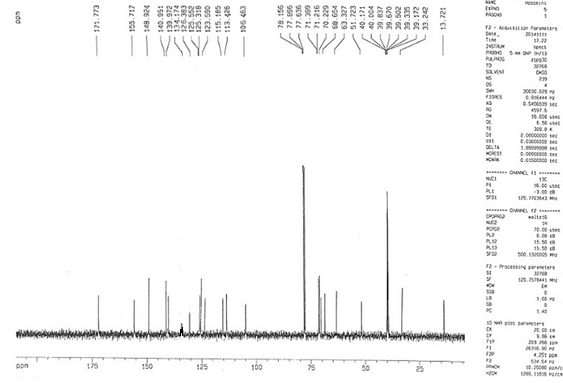

**Figure S-10.** ^13^C-NMR spectrum of flunixin meglumine **(6)** in CDCl_3_ & DMSO-*d_6_*


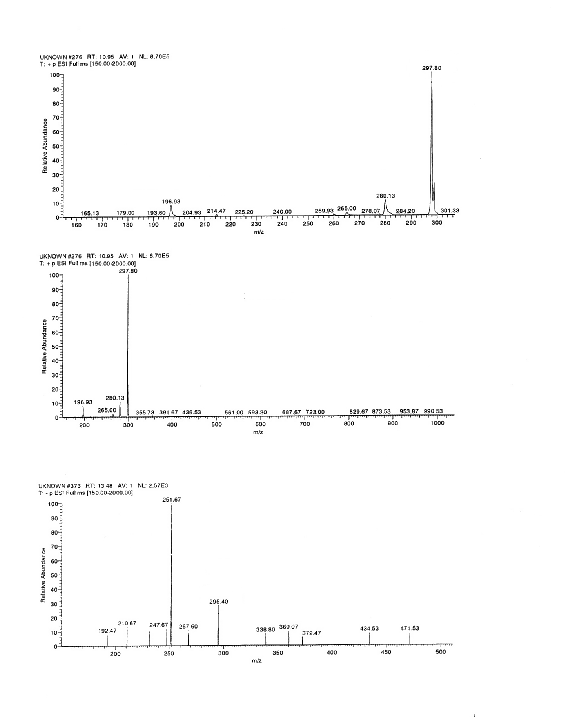

**Figure S-11.** Mass spectrum of flunixin meglumine **(6)**


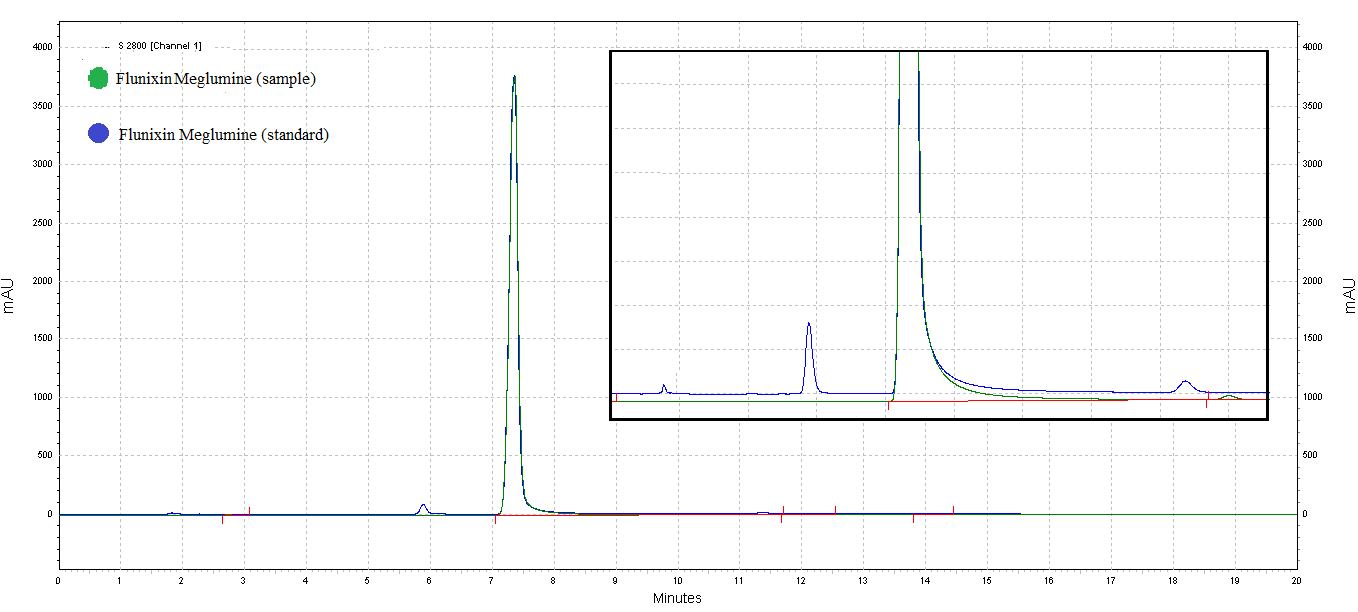


**Figure S-12.** Flunixin meglumine HPLC Chromatogram
